# Cytokines in Placental Physiology and Disease

**DOI:** 10.1155/2012/640823

**Published:** 2012-08-13

**Authors:** Dariusz Szukiewicz

**Affiliations:** Department of General and Experimental Pathology, Medical University of Warsaw, Ulica Żwirki i Wigury 61, 02-091 Warsaw, Poland

The placenta forms connection between growing fetus and uterine wall and is a unique, temporary, highly specialized organ required for the development of the embryo and fetus. In humans, as early as 3-4 days after fertilization trophoblastic cells can be identified on the outer layer of cells within the blastocyst. These precursor cells differentiate into all the other placental cell types that build the future, highly specialized organ. After approximately 12-13 weeks (end of the first trimester of gestation) development of the maternal blood supply to the placenta is completed. The most important functions and roles of the human placenta include metabolic (synthesis of glycogen, cholesterol, and fatty acids), transport and exchange within maternal-placental-fetal interface (gases, nutrients, and waste products), endocrine (synthesis and secretion of the pregnancy supporting hormones), immunological (immuno-suppressive function that protects the fetal “allograft” from T-cell-mediated immune aggression as well as—to some degree—protective role against infectious agents). 

Many research papers elucidated that physiological phenomena related to normal growth, development, and reproduction of living organisms are govern by complex cytokine interactions. It is becoming increasingly apparent that cytokines (CKs) not exclusively are synthesized within the “immune cells,” but are the product of a whole host of cell types, including nonimmune and/or structural cells such as epithelial cells, cytotrophoblast cells, and fibroblasts. Almost all CKs are pleiotropic effectors showing multiple biological activities. Interestingly, the human placenta produces a variety of CKs and expresses virtually all known CKs. Both successful placentation with trophoblast invasion and normal placental development accompanied by vascular and angiogenesis require precise balance between all members of the local (utero-placento fetal) cytokine network. 

Increased number of the innovative research approaches devoted to the better understanding of the connections between CKs, their receptors, and placental malfunction have recently been undertaken. On this background, the data about the role of cytokines in placental physiology and disease are still accumulating. The studies are focusing on the involvement of CKs in the processes of implantation, invasion of the trophoblast into the spiral uterine arteries, placental angiogenesis, response to inflammatory and immunologic factors in the uteroplacental interface, and induction of term/preterm labour. 

In this special issue of Mediators of Inflammation, I am pleased to present to the reader several articles written by experts in the field. All of them share the same subject mater, CKs in the human uteroplacental compartment. 

Modifications in the production of CKs by human placenta and fetal membranes are observed in response to infection-caused inflammation. However, precise changes in the placental cytokine profile during the course of an infection are hard to predict because of the heterogeneity of infectious agents. Extensive studies in this field, particularly related to the influenza virus infection, were performed by N. Uchide et al.

Recently, the results of significant studies on heritable changes in gene expression or cellular phenotype caused by mechanisms other than changes in the underlying DNA sequence have been published. The role of the so-called epigenetic factors may be greater than previously thought. Moreover, in contrast to genetically governed inheritance, epigenetic heritability is potentially reversible, which may facilitate implementation of new therapeutic strategies. The pathomechanism of preterm labour involves a shift in the balance between pro- and anti-inflammatory CKs synthesized by gestation tissues. Modulation of the placental cytokine production by epigenetic mechanisms has been proved. The original research paper dealing with the mentioned issues in the context of inhibitors of DNA methylation and a histone deacetylation is presented by M. D. Mitchell et al. It may be of high relevance, considering that immaturity remains a leading cause of neonatal morbidity and mortality worldwide.

A specific form of immunosuppression is required for a successful pregnancy, while protection of the fetus, which resembles a semiallogenic graft, should not be compromising for the mother. Depending on their pattern of cytokine synthesis, subpopulations Th1 and Th2 of T helper cells are distinguished. A shift from Th1 response towards Th2 profile was reported and proposed as the important mechanism of induction of maternal tolerance and suppression. An interesting review paper on the relationship between preterm labour and an aberrant Th1:Th2 profile is presented by L. Sykes et al. Elsewhere in this special issue, L. Sykes et al. published original paper on the influence of prostaglandin J_2_ (15dPGJ_2_) on the production of proinflammatory cytokines by T helper cells in pregnancy. Authors suggest potential therapeutic benefit of 15dPGJ_2_ in inflammation-induced preterm labour. 

Increased concentration of chemokine interleukin-8 (IL-8) has been demonstrated in the uterus during term and preterm labour. Another contribution to the knowledge of cytokine-dependent mechanisms that trigger contractile activity of the uterus was given by S. Khanjani et al. This work focuses on the roles of transcription factors (NF-*κ*B, AP-1, CEBP) in interleukin-1*β* (IL-1*β*) regulation of the IL-8 gene in myometrial cells cultured in vitro.

The pathogenesis of preeclampsia or pregnancy-induced hypertension remains largely unknown. It has been hypothesized that placental ischemia due to failed invasion of the trophoblastic cells in uterine spiral arteries may be an initiating factor. Different distribution of mast cells in the placenta and corresponding changes in histamine concentration may be involved in the defective placental vascularization seen in preeclampsia as reported by G. Szewczyk et al.

